# Supplementary material for: Viral introductions and return to baseline sexual behaviors maintain low-level mpox incidence in Los Angeles County, USA, 2023–2024
Source: medRxiv. 2025 Mar 15:2025.03.14.25323999. Preprint. [Version 1] doi: 10.1101/2025.03.14.25323999 (PMC11952628; doi:10.1101/2025.03.14.25323999)
Supplement: 1 [file NIHPP2025.03.14.25323999V1-supplement-1.pdf]

medRxiv preprint doi: https://doi.org/10.1101/2025.03.14.25323999; this version posted March 15, 2025. The copyright holder for this preprint (which was not certified by peer review) is the author/funder, who has granted medRxiv a license to display the preprint in perpetuity.
It is made available under a CC-BY 4.0 International license .

Supplementary Material:

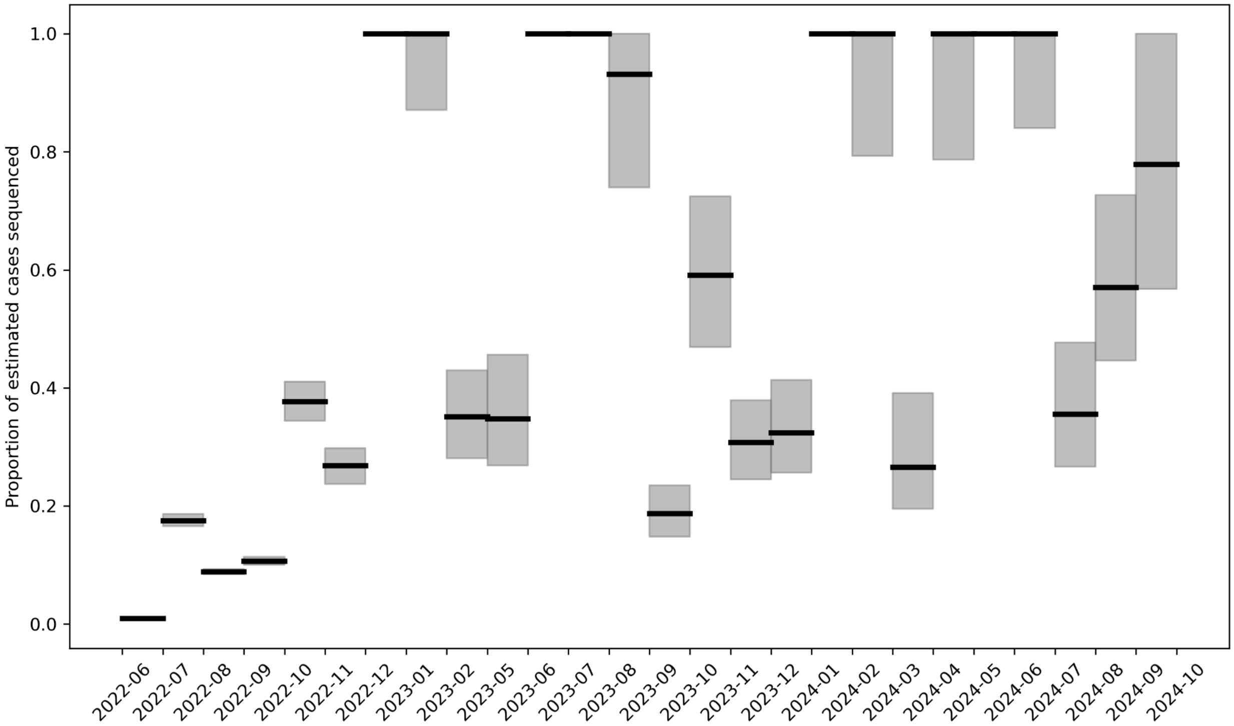

Figure S1: Estimated proportion of mpox cases sequenced in Los Angeles County. The proportion of cases sequenced was calculated by dividing the total number of mpox sequences from LAC found on GenBank by the monthly mpox incidence estimated from case counts using a renewal equation framework. The dark black horizontal line refers to the median estimates with the grey bars representing

medRxiv preprint doi: https://doi.org/10.1101/2025.03.14.25323999; this version posted March 15, 2025. The copyright holder for this preprint (which was not certified by peer review) is the author/funder, who has granted medRxiv a license to display the preprint in perpetuity.
It is made available under a CC-BY 4.0 International license .

the 95% CI based on uncertainty in the incidence estimates. Months where the estimated proportion was greater than 100% (due to uncertainty incidence estimation due to low case counts or sample collection at dates different than diagnosis) were bounded at 100%.

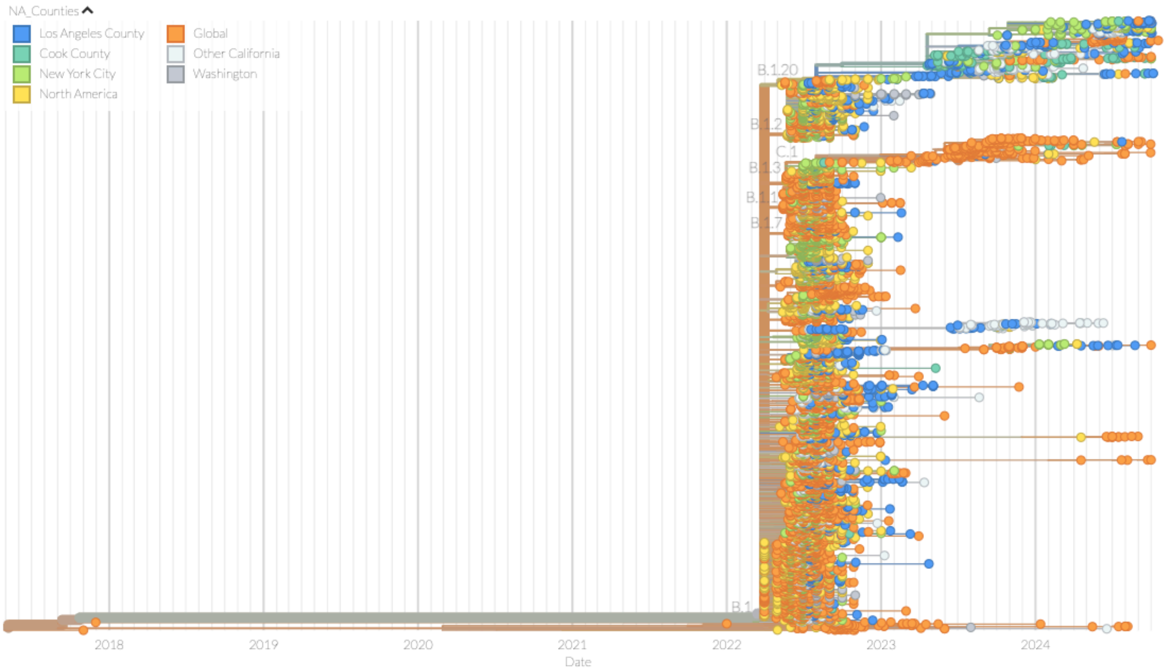

Figure S2: Time-resolved maximum likelihood phylogenies for mpox clade IIb sample collection dates between January 1 2022 and December 12, 2024 Tip color represents the focus areas in North America with high sequencing effort. Branches are colored based on inferred ancestry. The full tree can be explored interactively at https://nextstrain.org/groups/blab/mpox-la/allcladeIIseqs?c=focus_areas

medRxiv preprint doi: https://doi.org/10.1101/2025.03.14.25323999; this version posted March 15, 2025. The copyright holder for this preprint (which was not certified by peer review) is the author/funder, who has granted medRxiv a license to display the preprint in perpetuity.
It is made available under a CC-BY 4.0 International license .

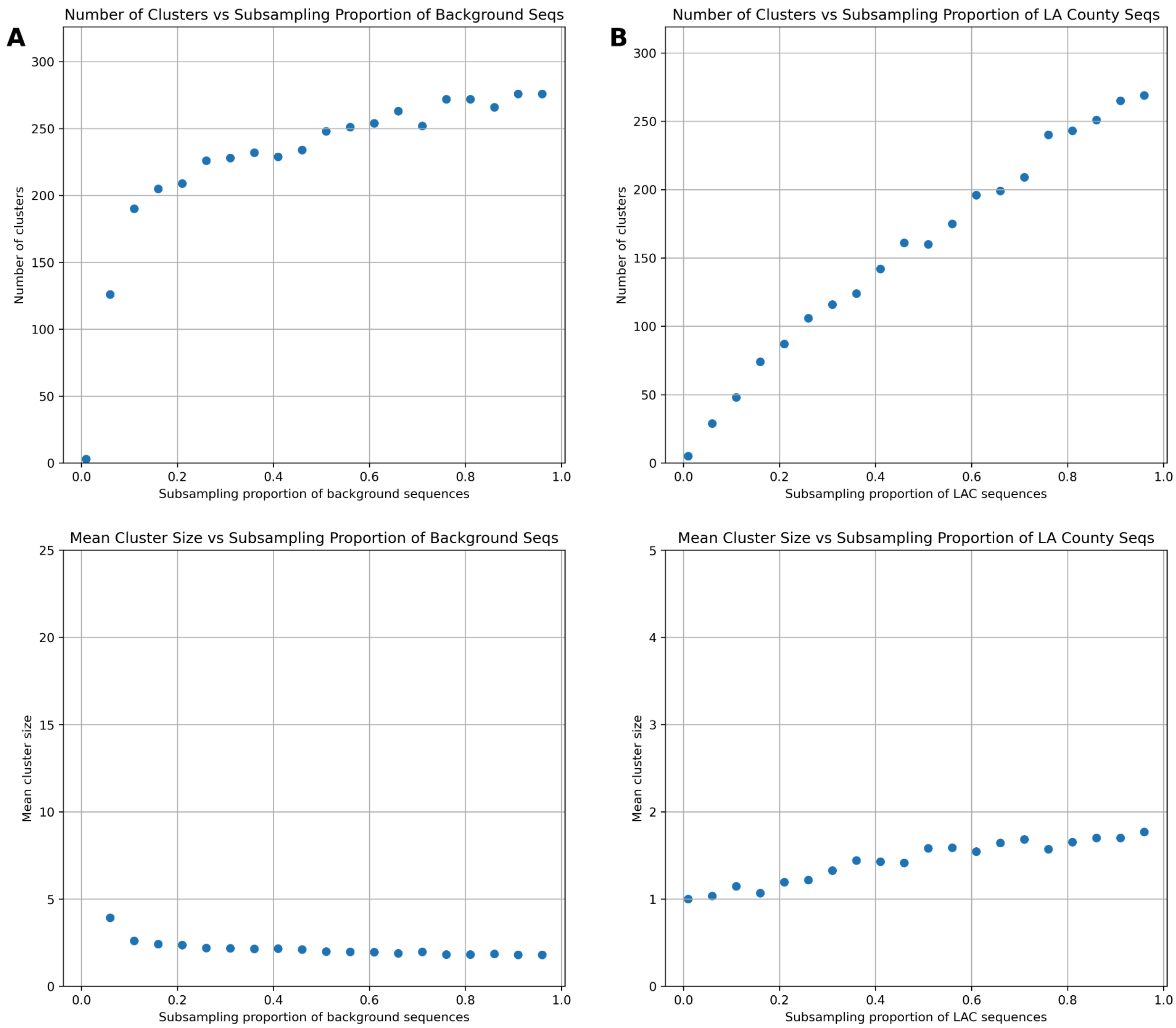

Figure S3: The impact of subsampling on the number and size of transmission clusters identified. We randomly subsampled different proportions of background sequences **(A)** and sequences from LA **(B)** and ran our clustering algorithm to show the impact of increasing the proportion sequences relative to the full dataset on the total number of clusters identified (Top row) and the mean size of those clusters (bottom row).

medRxiv preprint doi: https://doi.org/10.1101/2025.03.14.25323999; this version posted March 15, 2025. The copyright holder for this preprint
(which was not certified by peer review) is the author/funder, who has granted medRxiv a license to display the preprint in perpetuity.
It is made available under a CC-BY 4.0 International license .

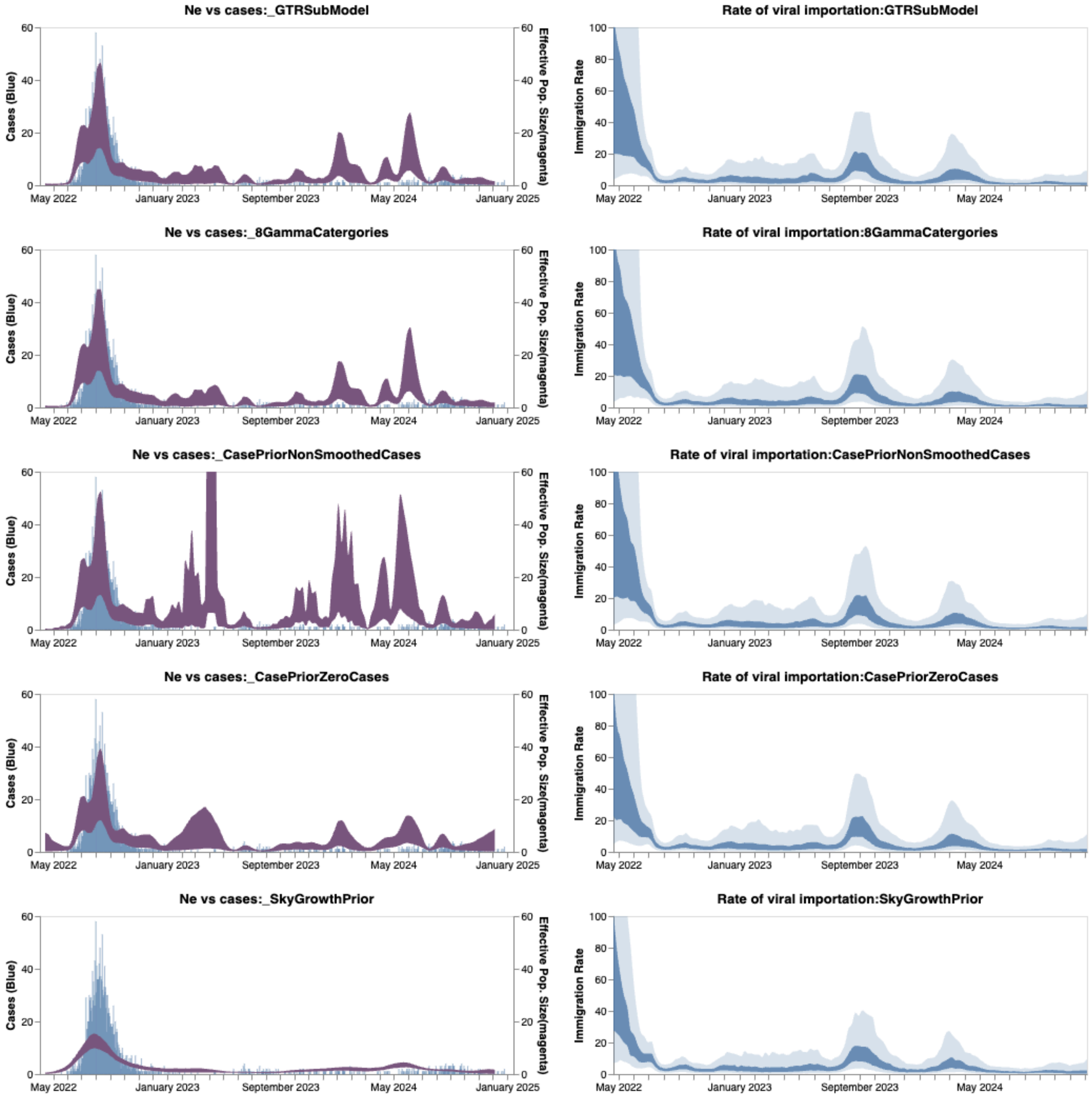

Figure S4: Sensitivity analysis of phylodynamic results: We tested the robustness of our phylodynamic results by repeating our main analyses under different model specifications. The left column shows the empirical case counts in blue and the estimated effective population size (Ne) in magenta (50% HPD). The right columns the inferred immigration rate of mpox into LAC, with the dark blue band representing the 50% HPD and the lighter blue representing the 95% HPD. The first row represents the same case-informed estimates as our main result but with a GTR substitution model instead of HKY. Second row represents 8 category discretization of the gamma distribution prior instead of 4 categories. The third row is our main model but without the cases being smoothed prior to being inputted into the model. The fourth row is a skyline prior represented by having zero case information in the case prior. The final row is a skygrowth prior with no case information instead of the skyline case-informed prior.

medRxiv preprint doi: https://doi.org/10.1101/2025.03.14.25323999; this version posted March 15, 2025. The copyright holder for this preprint
(which was not certified by peer review) is the author/funder, who has granted medRxiv a license to display the preprint in perpetuity.
It is made available under a CC-BY 4.0 International license .

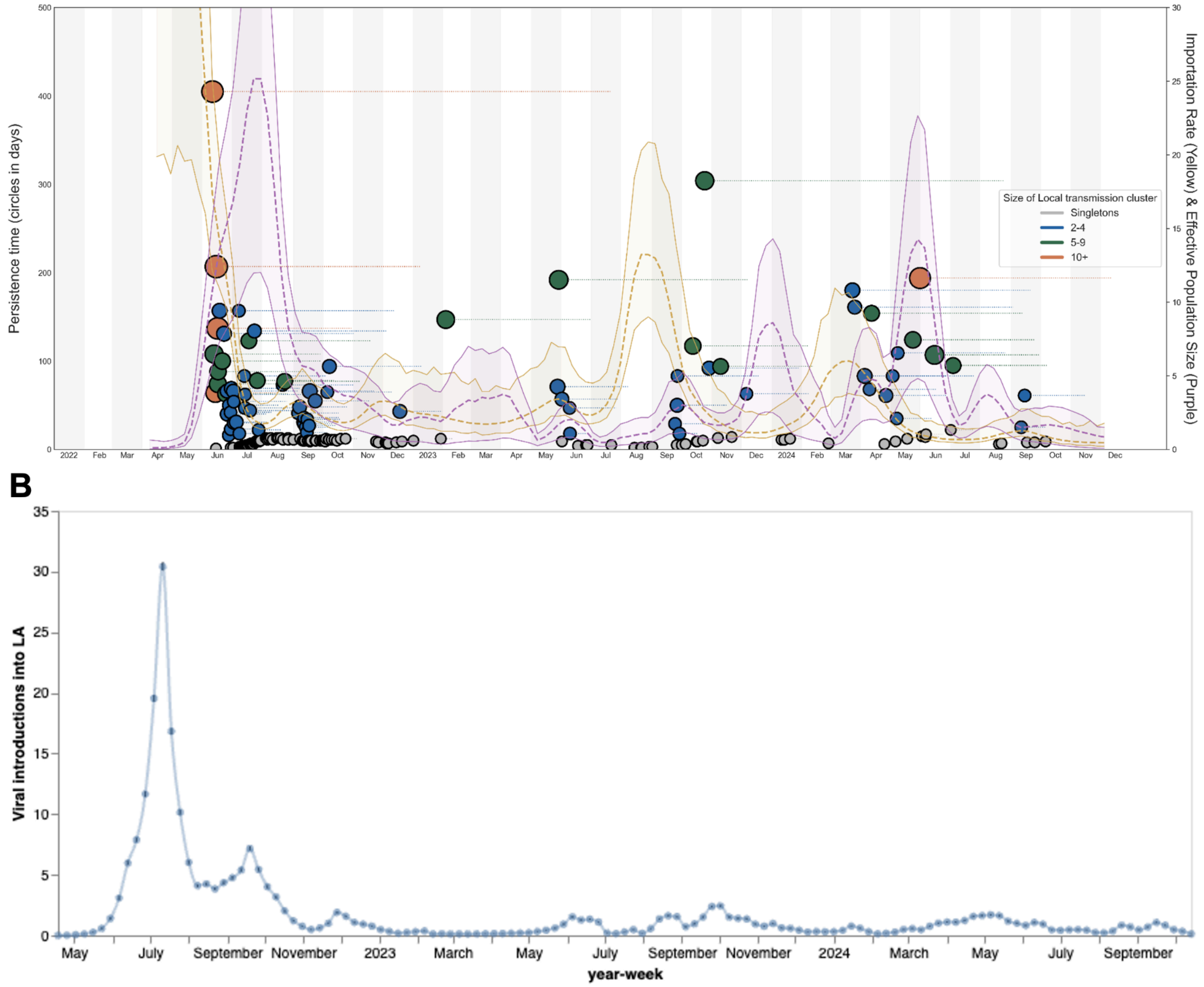

Figure S5: Mpox importation dynamics in LA County estimated via Bayesian Phylodynamics. Panel **A** shows the persistence time of each identified local outbreak cluster according to the date of its inferred introduction time. Each dot represents an inferred introduction into LA County, the radius of the dot is proportional to the size of the resulting transmission cluster. The yellow streamgraph is the rate of introduction (events/lineage/year) into LA county and the purple streamgraph represents the estimated effective population size**,** both inferred by our phylodynamic model. The dashed line represents the median and the bands represent the 95% HPD. Panel **B** shows the absolute number of viral introductions inferred via our phylodynamic model for each week that was calculated by analyzing the entire posterior set of phylogenetic trees after burn in. The error bars represent the 95% CI and these estimates were used to parameterize our microsimulation model.

medRxiv preprint doi: https://doi.org/10.1101/2025.03.14.25323999; this version posted March 15, 2025. The copyright holder for this preprint (which was not certified by peer review) is the author/funder, who has granted medRxiv a license to display the preprint in perpetuity.
It is made available under a CC-BY 4.0 International license .

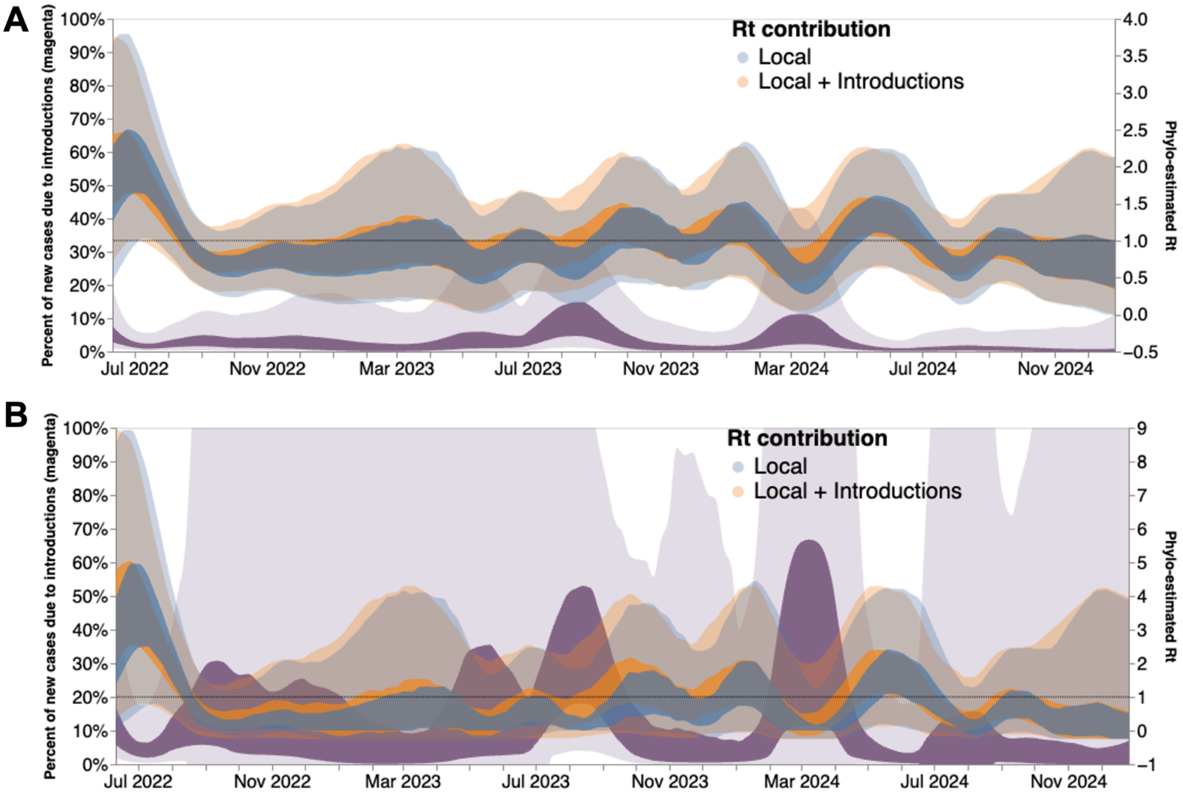

Figure S6: Percentage of new cases due to introductions and Rt with infectious period of (A) 4.5 and (B) 21 days. The inner area denotes the 50% HPD interval, and the outer area denotes the 95% HPD interval. The blue and orange bands lines represent estimates of Rt highlighting the contribution of local transmission only (blue) as well as that of viral introductions (orange). Dashed line highlights an Rt value of 1. Rt estimates were smoothed using a 14-day rolling average.

medRxiv preprint doi: https://doi.org/10.1101/2025.03.14.25323999; this version posted March 15, 2025. The copyright holder for this preprint (which was not certified by peer review) is the author/funder, who has granted medRxiv a license to display the preprint in perpetuity.
It is made available under a CC-BY 4.0 International license .

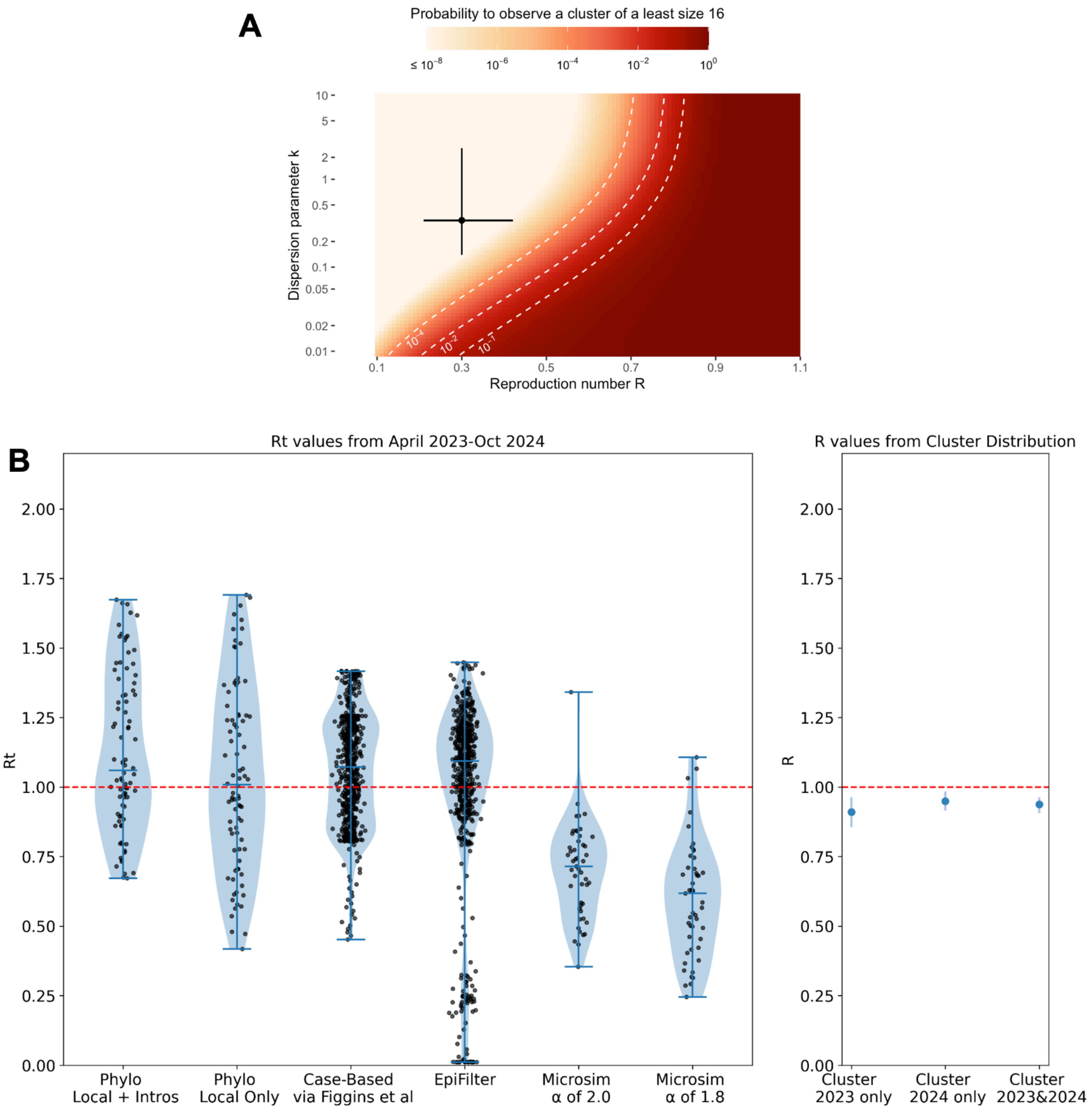

Figure S7: Reproductive number estimation for LAC from March 2023 through October 2024. A. Probability to observe a cluster of size 16 among 64 clusters as a function of the reproduction number R and the dispersion parameter k assuming 5.5% of infections are sequenced. The horizontal and vertical lines correspond to estimates obtained by Blumberg and Lloyd-Smith (26) from the analysis of epidemiological clusters during previous outbreaks. The dotted white lines correspond to contour lines for probabilities of 10−4, 10−2, and 10−1. **B.** The mean estimates of Rt (left) or R (right) for mpox showing the spread via a violin plot with the extremes and the median highlighted by the darker blue horizontal lines. The left panel plots the spread of weekly Rt estimations while the right panel shows the estimates of R with 95% CIs found from the distribution of cluster sizes for either 2023, 2024, or both years combined. The x axis of the left panel shows the methodology used and the dashed red line denotes an R or Rt of 1.

medRxiv preprint doi: https://doi.org/10.1101/2025.03.14.25323999; this version posted March 15, 2025. The copyright holder for this preprint (which was not certified by peer review) is the author/funder, who has granted medRxiv a license to display the preprint in perpetuity.
It is made available under a CC-BY 4.0 International license .

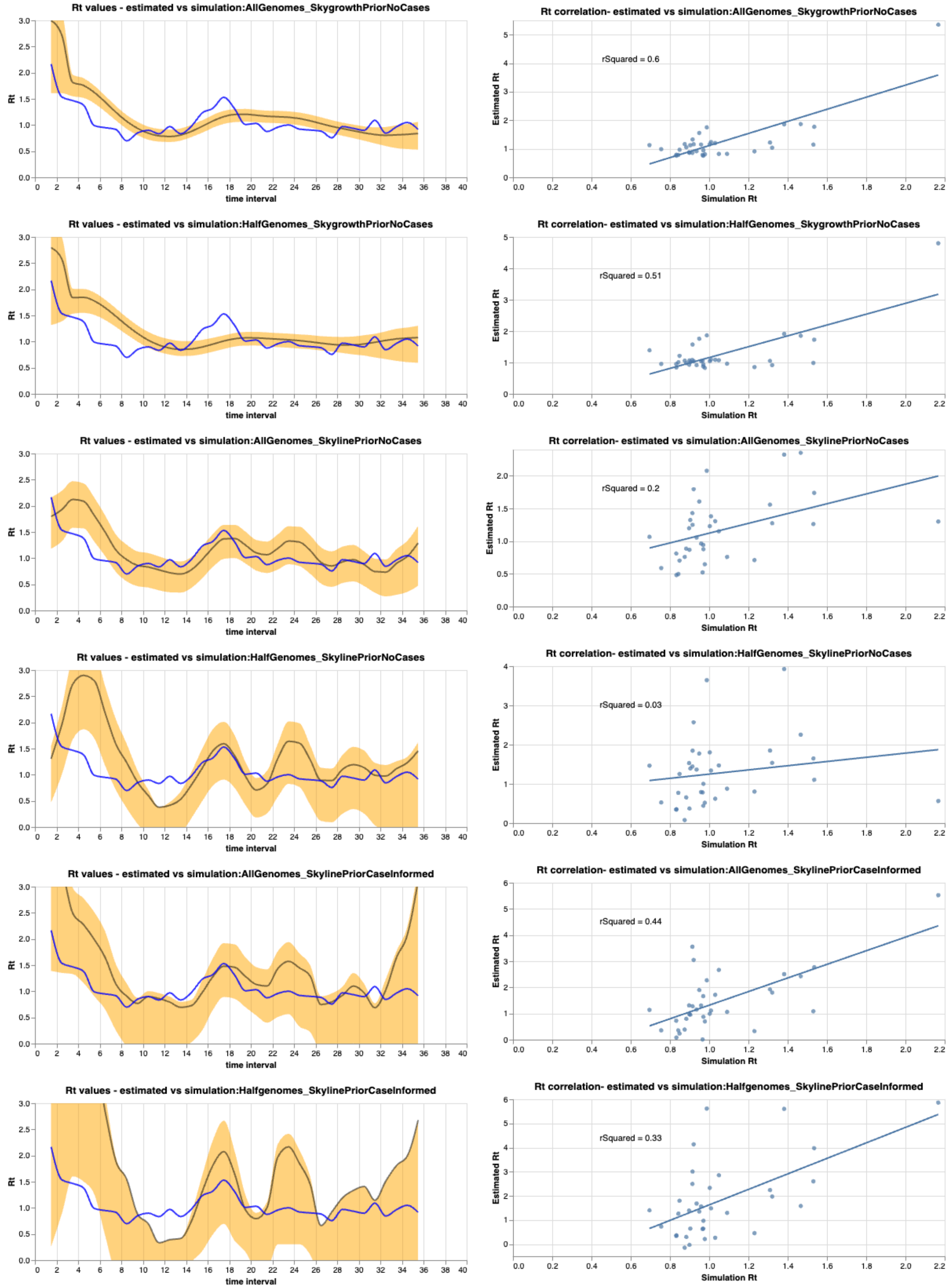

Figure S8: Estimation of Rt from simulations. We tested the ability of our multitree coalescent approach under different Ne priors to correctly estimate Rt from simulations. To do so, we simulated a

medRxiv preprint doi: https://doi.org/10.1101/2025.03.14.25323999; this version posted March 15, 2025. The copyright holder for this preprint (which was not certified by peer review) is the author/funder, who has granted medRxiv a license to display the preprint in perpetuity.
It is made available under a CC-BY 4.0 International license .

local outbreak using a constant rate of introduction. We then sampled cases based on the estimated time to seek care during the 2022 mpox epidemic, subsampled, simulated genetic sequences, and then used the local transmission cluster to estimate Rt. The left column shows the true Rt in blue with the estimated Rt in yellow (showing the 95% HPD intervals) with the grey line representing the median estimate. The right column shows the correlation between simulated and the estimated Rt with a linear regression fit and R^2 calculated. The first two rows represent a skygrowth prior on growth rate, the second two represent a skyline prior on the Ne without cases, and the bottom set of two represent the main analysis of a skyline prior on the Ne informed by mpox cases. For each set of priors, the top analysis represents 100% of the sampled genomes used while the bottom analysis represents only 50% of the genomes used. Estimates were smoothed using a 14 day rolling average.

medRxiv preprint doi: https://doi.org/10.1101/2025.03.14.25323999; this version posted March 15, 2025. The copyright holder for this preprint (which was not certified by peer review) is the author/funder, who has granted medRxiv a license to display the preprint in perpetuity.
It is made available under a CC-BY 4.0 International license .

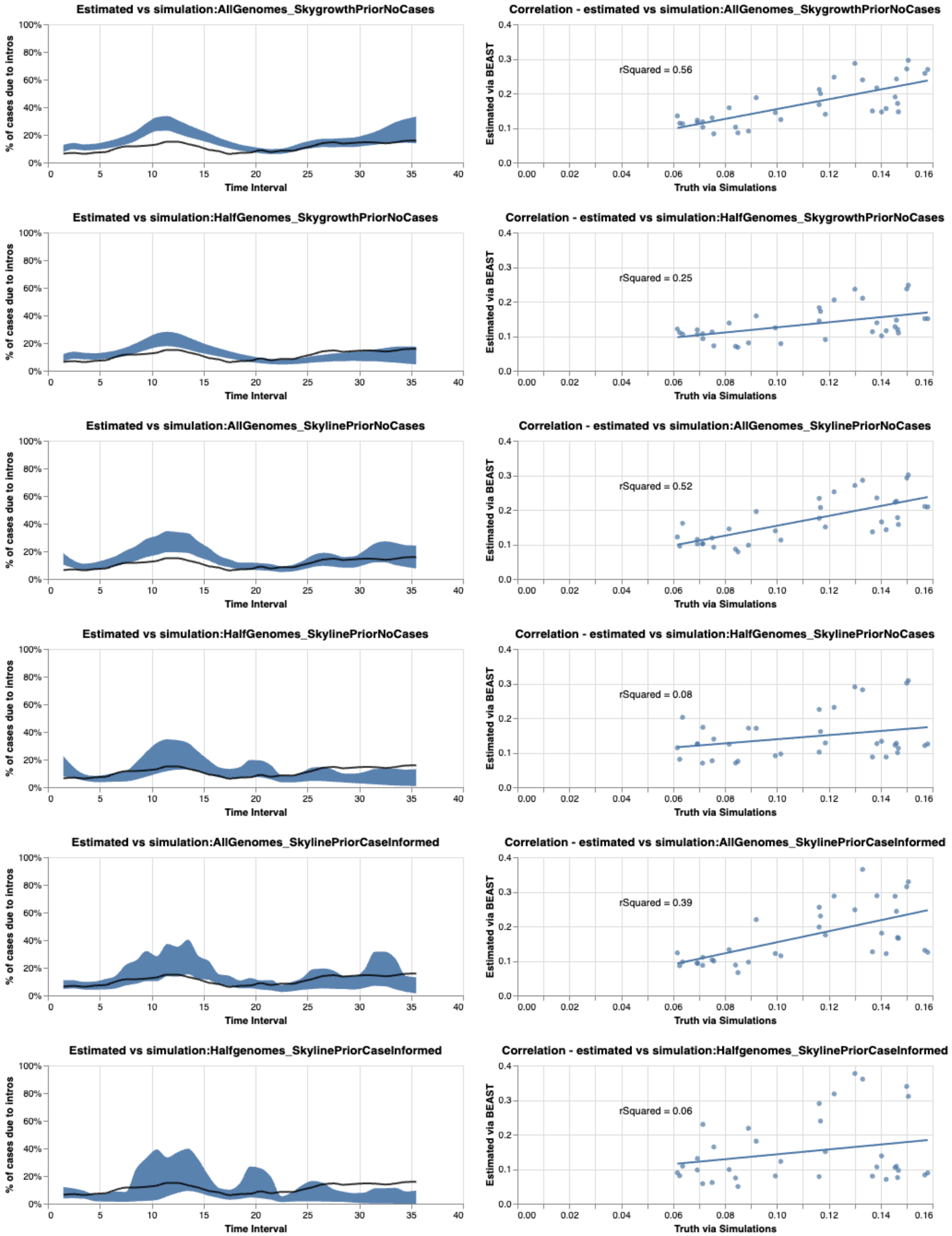

Figure S9: Estimation of percentage of cases due to introductions from simulations. We tested the ability of our multitree coalescent approach under different Ne priors to correctly estimate the percentage of cases due to introductions from simulations. To do so, we simulated a local outbreak using a constant

medRxiv preprint doi: https://doi.org/10.1101/2025.03.14.25323999; this version posted March 15, 2025. The copyright holder for this preprint (which was not certified by peer review) is the author/funder, who has granted medRxiv a license to display the preprint in perpetuity.
It is made available under a CC-BY 4.0 International license .

rate of introduction. We then sampled cases based on the estimated time to seek care during the 2022 mpox epidemic, subsampled, simulated genetic sequences, and then used the local transmission cluster to estimate percentage The left column shows the true percentage in blue with the estimated percentage in yellow (showing the 95% HPD intervals) with the grey line representing the median estimate. The right column shows the correlation between simulated and the estimated percentage with a linear regression fit and R^2 calculated. The first two rows represent a skygrowth prior on growth rate, the second two represent a skyline prior on the Ne without cases, and the bottom set of two represent the main analysis of a skyline prior on the Ne informed by mpox cases. For each set of priors, the top analysis represents 100% of the sampled genomes used while the bottom analysis represents only 50% of the genomes used. Estimates were smoothed using a 14 day rolling average.

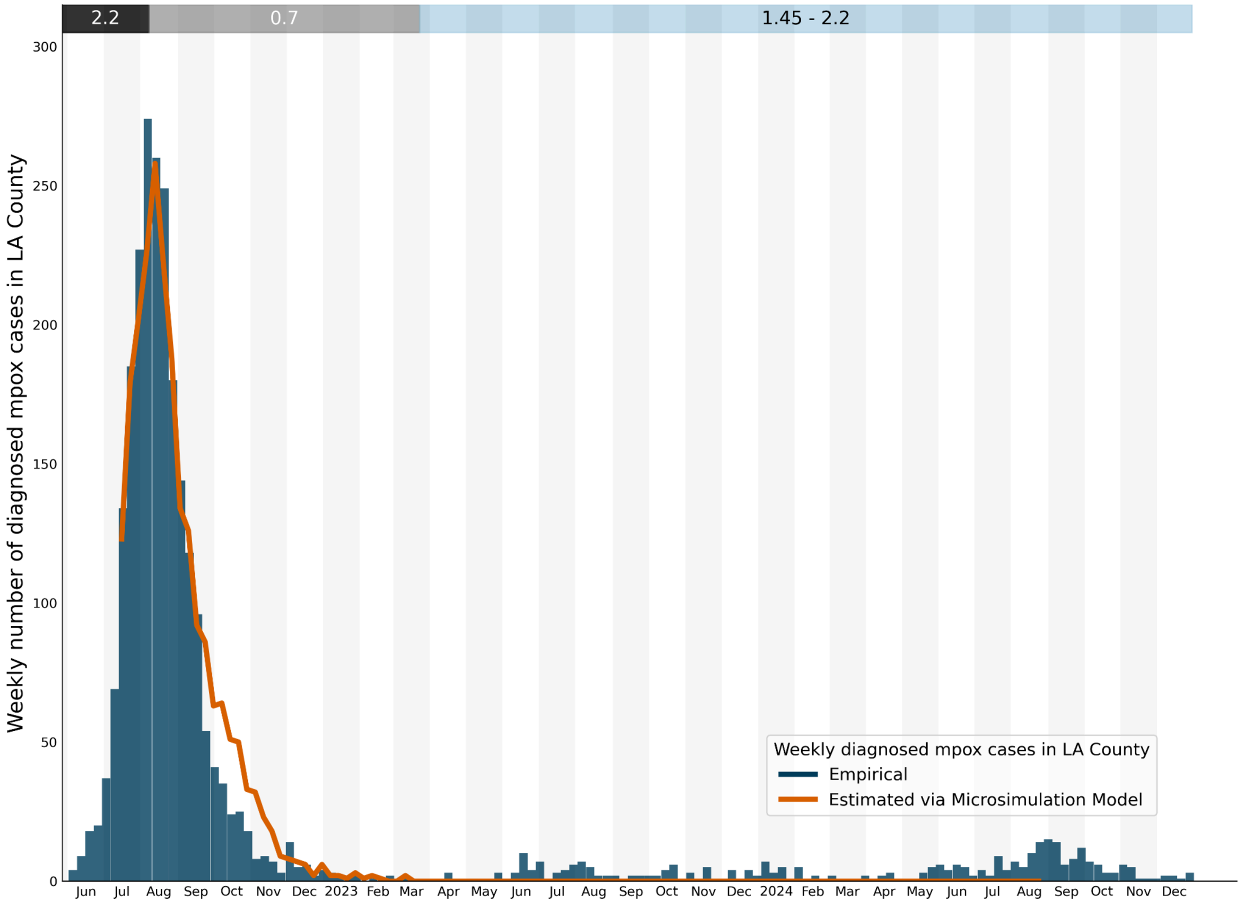

Figure S10: Infectivity Scalar (α) during 2022 mpox outbreak in Los Angeles County. Main figure shows the weekly number of diagnosed mpox cases in LAC from June 2022 through December 2024 (blue) with the number of diagnosed mpox cases simulated via our microsimulation model without viral importations overlaid in orange. The bars in the top of the figure are a visual representation of the periods of time for which α was calibrated. The grey bars represent the initial model calibration for the epidemic period and the blue bar shows the period of interest for this study.

medRxiv preprint doi: https://doi.org/10.1101/2025.03.14.25323999; this version posted March 15, 2025. The copyright holder for this preprint (which was not certified by peer review) is the author/funder, who has granted medRxiv a license to display the preprint in perpetuity.
It is made available under a CC-BY 4.0 International license .

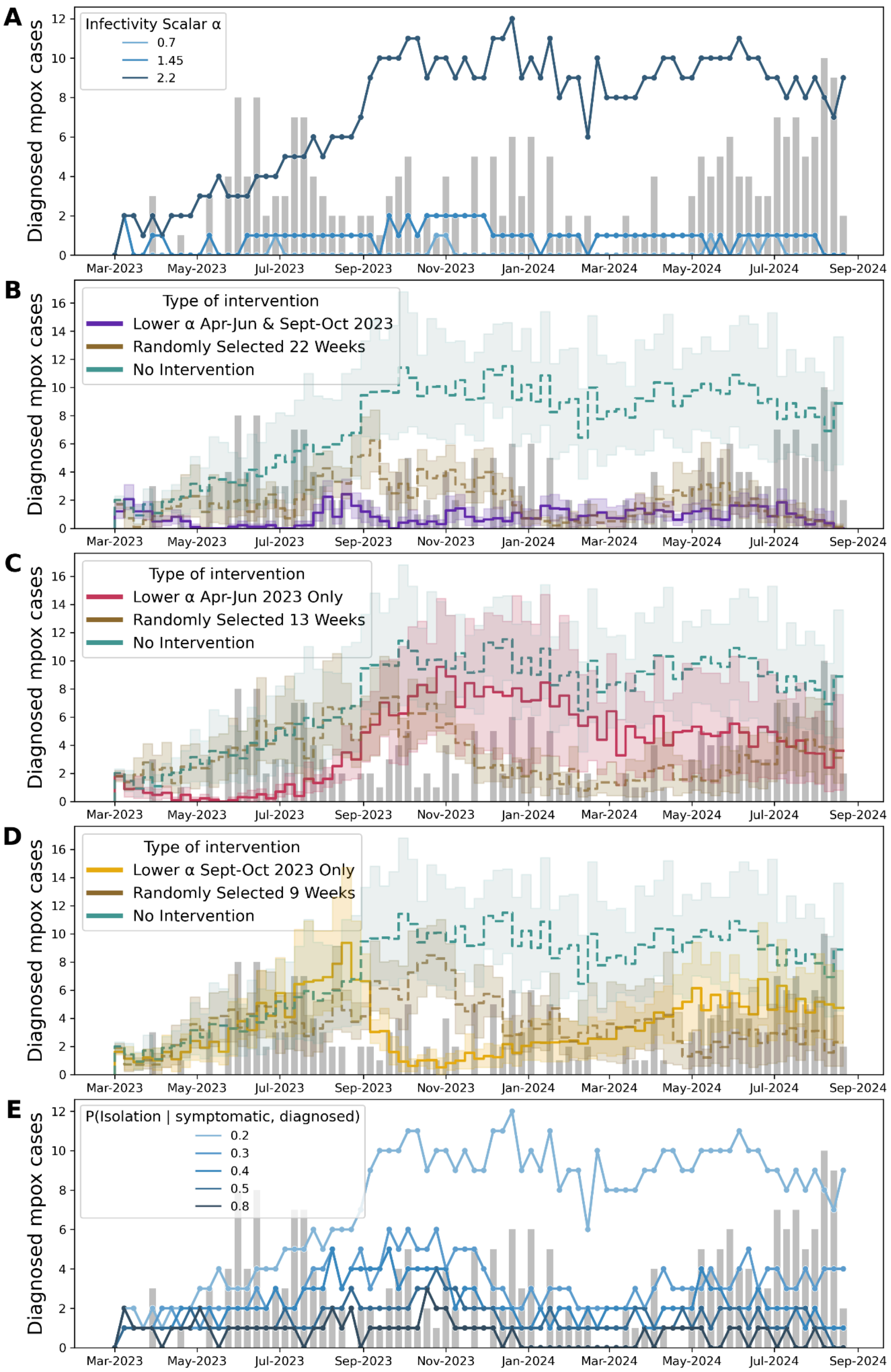

Figure S11: Factors maintaining mpox prevalence and modeling counterfactual public health interventions with statistical tests and without uncertainty intervals. (A) we explored the Infectivity Scalar α that best explains the empirical weekly number of diagnosed mpox cases (gray bars). Line graphs represent the mean weekly number of mpox diagnoses simulated using increasing α. **(B-D)** Given

medRxiv preprint doi: https://doi.org/10.1101/2025.03.14.25323999; this version posted March 15, 2025. The copyright holder for this preprint (which was not certified by peer review) is the author/funder, who has granted medRxiv a license to display the preprint in perpetuity.
It is made available under a CC-BY 4.0 International license .

the non-constant pattern of viral introductions seen in the phylodynamic analysis, we tested different counterfactual scenarios of public health interventions during specific time periods represented by lowering the α to 0.7 while keeping the α at 2.2 during the remaining time. The bold yellow, red, and purple solid lines represent the simulated weekly number of diagnosed mpox cases under phylodynamic-informed interventions. To test for non-specific effects, we also reran our microsimulation model by randomly selecting the same number of weeks as our phylodynamics-informed interventions to lower the α to 0.7 (brown dashed lines) as well as a simulation without any interventions. (green dashed lines) **(C)** We also tested the effect of increasing the probability of isolating upon a symptomatic individual receiving a positive mpox diagnosis on the simulated number of diagnosed mpox cases (blue line graphs). In all plots, the grey bars represent the empirical number of mpox diagnoses in LAC. We switch to line plots instead of step plots and remove the uncertainty intervals in **A** and **E** to allow easier visualization of overlapping lines.

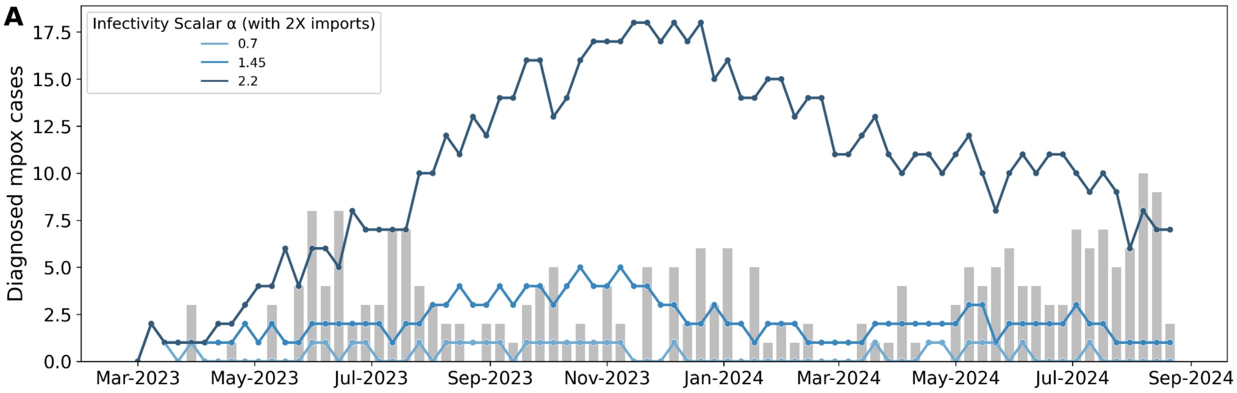

Figure S12: Infectivity Scalar α with twice as many phylodynamics-informed viral introductions. To test the impact of underestimating the number of viral introductions into LAC, we doubled the number of introductions, reran our microsimulation model, and explored the Infectivity Scalar α that best explains the empirical weekly number of diagnosed mpox cases (gray bars). Line graphs represent the mean weekly number of mpox diagnoses simulated using increasing α. Each weekly estimate represents the average of 10 independent iterations of our model

medRxiv preprint doi: https://doi.org/10.1101/2025.03.14.25323999; this version posted March 15, 2025. The copyright holder for this preprint
(which was not certified by peer review) is the author/funder, who has granted medRxiv a license to display the preprint in perpetuity.
It is made available under a CC-BY 4.0 International license .

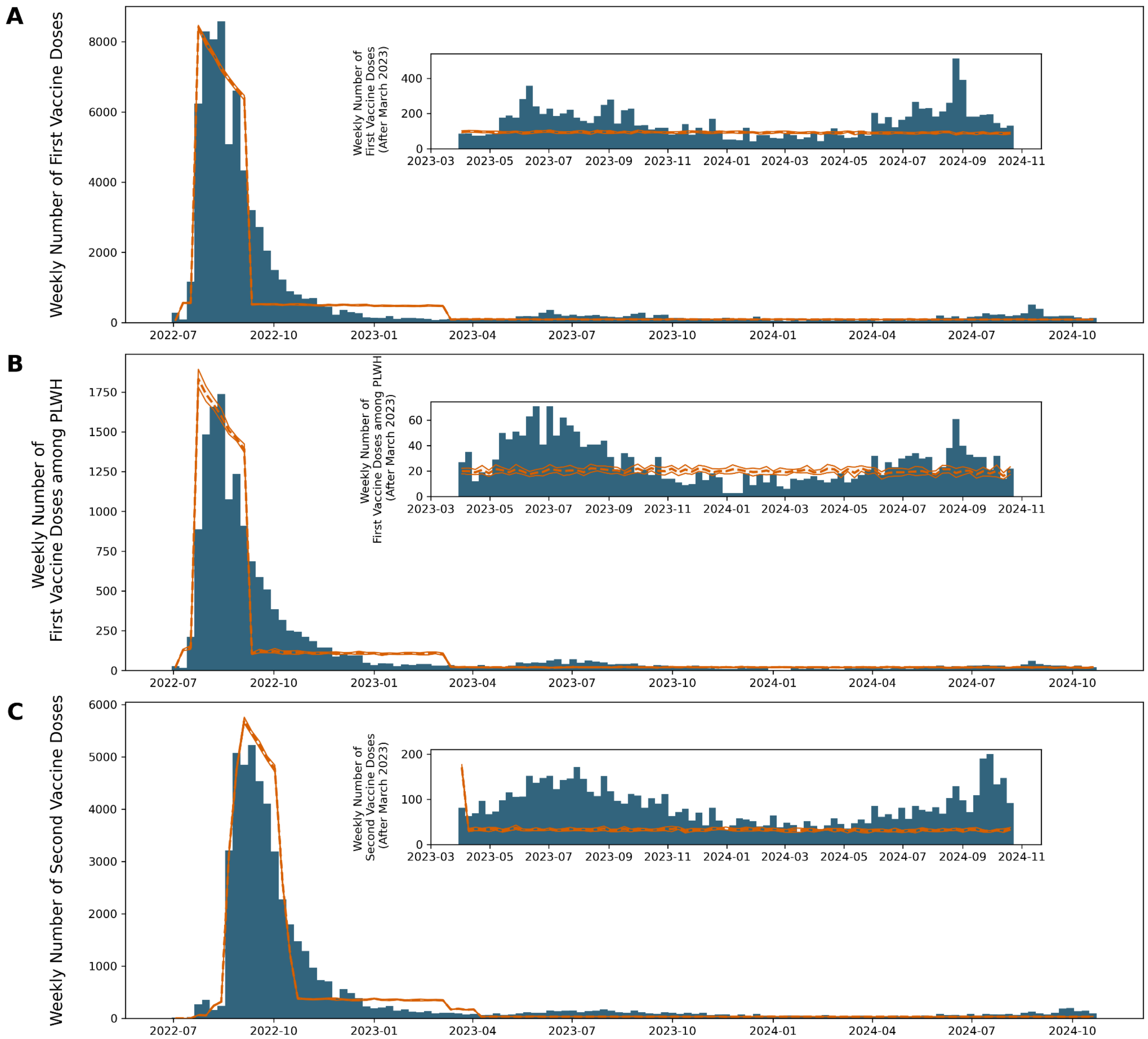

Figure S13: Vaccination validation of microsimulation model. All three panels represent the comparison between the empirical number of vaccination doses given (dark blue) and the number of doses administered as simulated by our model (orange). The dashed orange line represents the mean; the bands represent the 95% uncertainty interval calculated via bootstrapping. The inset graphs for each panel represents the same data but only after March 2023 to allow for better visualization of smaller numbers. Panel **A** represents the comparison of the number of first doses of the mpox vaccine given, panel **B** is for the number of first doses among people living with HIV (PLWH), and panel **C** is the number of second doses given.
